# Comparative Transcriptome Analysis Reveals the Molecular Immunopathogenesis of Chinese Soft-Shelled Turtle (*Trionyx sinensis*) Infected with *Aeromonas hydrophila*

**DOI:** 10.3390/biology10111218

**Published:** 2021-11-22

**Authors:** Zhao Lv, Yazhou Hu, Jin Tan, Xiaoqing Wang, Xiaoyan Liu, Cong Zeng

**Affiliations:** 1 Hunan Engineering Technology Research Center of Featured Aquatic Resources Utilization, Hunan Agricultural University, Changsha 410128, China; lvzhao_0320@hunau.edu.cn (Z.L.); huyazhou@hunau.edu.cn (Y.H.); tan775481248@163.com (J.T.); wangxiao@hunau.net (X.W.); liuxy86@163.com (X.L.); 2 School of Oceanography, Shanghai Jiao Tong University, Shanghai 200240, China

**Keywords:** Chinese soft-shelled turtle, *Aeromonas hydrophila*, hemorrhagic sepsis, molecular immunopathogenesis

## Abstract

**Simple Summary:**

The Chinese soft-shelled turtle (*Trionyx sinensis*) is an important cultured reptile in East Asia. Hemorrhagic sepsis caused by *Aeromonas hydrophila* infection is the dominant disease in the aquaculture of Chinese soft-shelled turtles, while the molecular pathology is far from clear due to the lag of research on turtle immunology. It has been reported in mammals and fish that the dysfunction of immune responses to pathogen infections causes host tissue hemorrhagic sepsis. In this study, two groups of turtles with different susceptibility to *A. hydrophila* infection are found. A comparative transcriptome strategy is adopted to examine the gene expression profiles in liver and spleen for these two phenotypes of turtles post *A. hydrophila* infection, for the first time revealing the full picture of immune mechanisms against *A. hydrophila*, which provides new insight into the molecular pathology during *A. hydrophila* infection in *T. sinensis*. The findings will promote further investigations on pathogenic mechanisms of hemorrhagic sepsis caused by *A. hydrophila* infection in *T. sinensis*, and also will benefit their culture industry.

**Abstract:**

Although hemorrhagic sepsis caused by *Aeromonas hydrophila* infection is the dominant disease in the aquaculture of Chinese soft-shelled turtle, information on its molecular pathology is seriously limited. In this study, ninety turtles intraperitoneally injected with *A. hydrophila* exhibited two different phenotypes based on the pathological symptoms, referred to as active and inactive turtles. Comparative transcriptomes of liver and spleen from these two groups at 6, 24, and 72 h post-injection (hpi) were further analyzed. The results showed that cytokine–cytokine receptor interaction, PRRs mediated signaling pathway, apoptosis, and phagocytosis enriched in active and inactive turtles were significantly different. Pro-inflammatory cytokines, the TLR signaling pathway, NLR signaling pathway, and RLR signaling pathway mediating cytokine expression, and apoptosis-related genes, were significantly up-regulated in inactive turtles at the early stage (6 hpi). The significant up-regulation of phagocytosis-related genes occurred at 24 hpi in inactive turtles and relatively lagged behind those in active turtles. The anti-inflammatory cytokine, IL10, was significantly up-regulated during the tested periods (6, 24, and 72 hpi) in active turtles. These findings offer valuable information for the understanding of molecular immunopathogenesis after *A. hydrophila* infection, and facilitate further investigations on strategies against hemorrhagic sepsis in Chinese soft-shelled turtle *T. sinensis*.

## 1. Introduction

Chinese soft-shelled turtle (*Trionyx sinensis*) is an important reptile in East Asia and has been taken as a food resource for a long time in these areas, especially in China and Japan. In ancient Chinese medicine descriptions, consumption of turtles may bring about positive effects for human health, including strengthening immunity, anti-aging, and curing cardiocerebrovascular diseases [1]. The excellent nutriment and medical values mean the turtles have become one of the most critical freshwater aquaculture reptiles, and the annual production is over 325,000 tons in China. However, infectious diseases caused by bacteria and viruses have resulted in severe losses to the aquaculture of turtles [2,3]. It is noteworthy that there are more than 15 kinds of diseases caused by *Aeromonas hydrophila* infection, such as red neck disease, septicemia, furunculosis, etc., accounting for about 60% of the total disease cases in turtles [4,5]. The histopathology of Chinese soft-shelled turtles infected by *A. hydrophila* has been previously described. In general, the pathogenic processes undergo the adhesion of bacteria adhesion factors, and the destruction of host liver, respiratory, and digestive organs by virulence factors such as exotoxin and extracellular enzymes, which finally leads to serious tissue hemorrhagic sepsis and the death of turtles [6,7]. Nevertheless, the molecular pathology of hemorrhagic sepsis caused by *A. hydrophila* infection is far from being elucidated in Chinese soft-shelled turtles.

It has been reported in mammals and fish that the dysfunction of host immune responses to pathogen infections causes tissue hemorrhagic sepsis [8,9,10]. The immune system generally serves as the security guard for hosts, which holds an immune network composed of many immune cells [11]. The cells of the innate immune system consisting of neutrophils, monocytes, macrophages, dendritic cells, and natural killer cells recognize pathogens, produce cytokines, and engulf pathogens through phagocytosis, which are the first line of host defense against pathogens [12]. These innate immune cells rely on pattern recognition receptors (PRRs) including toll-like receptors (TLRs), nucleotide-binding domain (NOD)-like receptors (NLRs), and retinoic acid-inducible gene I-like receptors (RLRs) to recognize various microbial invaders and produce cytokines that further activate the innate as well as adaptive immune cells [13]. Under normal immune response, immune cells moderately produce and release cytokines, including interleukins (ILs), chemokines, and tumor necrosis factors (TNFs), which can contact more immune cells to participate in the battle between the host and foreign pathogens [14]. When immune cells produce excessive cytokines, especially pro-inflammatory cytokines such as IL1, IL6, IL18, and TNFs (“cytokine storm”), the host immune system is overactivated and attacks self-tissues or cells, causing systemic inflammation, tissue hemorrhagic sepsis, and even death [15,16].

Although the research of turtle immunology lags behind that of mammals and fish, the unique evolutionary status as secondary aquatic reptiles has recently aroused wide concern on the distinct immune response mechanism against pathogens in turtles [17,18,19,20]. Fifteen candidate TLR family genes have been identified in *T. sinensis* [21]. After *A. hydrophila* infection, TLR2 and TLR4 are significantly up-regulated in the spleen, indicating the immune response of TLR signaling pathway during bacterial infection in *T. sinensis* [21]. Zhou et al. identified an IL8 homolog from *T. sinensis* and confirmed that IL8 mRNA expression shows significant up-regulation in various tissues, including liver, spleen, kidney, heart, intestine, and blood, after *A. hydrophila* infection [22]. Zhang et al. also investigated the mRNA expression changes of pro-inflammatory cytokines such as IL1β, TNFα, IL6, IL8, and IL12 in *T. sinensis* during acute cold stress, revealing that acute cold stress increases the expression of pro-inflammatory cytokines in the spleen and intestine to withstand *A. hydrophila* infection [23]. These above studies may help us understand the mRNA expression profiles and function of several immune molecules in *T. sinensis*. However, the evidence on the immune response mechanism in *T. sinensis* so far is too scattered and limited, which largely hinders the studying of pathogen–host immunity interaction and the molecular pathology during *A. hydrophila* infection in Chinese soft-shelled turtles.

RNA-sequencing (RNA-Seq) using next-generation sequencing is one of the most useful methods to survey the character of a transcriptome because it offers the whole data on gene expression. To date, transcriptome profiling using next-generation sequencing technologies has provided new insights into pathogen–host immunity interaction in many aquaculture fish species, such as Nile tilapia (*Oreochromis niloticus*) [24], Gold fish (*Carassius auratus* L.) [25], Ussuri catfish (*Pseudobagrus ussuriensis*) [26], Yellow catfish (*Pelteobagrus fulvidraco*) [27], and Atlantic salmon (*Salmo salar*) [28]. More and more studies have used this high-throughput sequencing approach to identify the expression differences of immune molecules between the resistant and susceptible fish to pathogen infection. For example, Moraleda et al. analyzed the comparative transcriptomes of resistant/susceptible salmons with different immune responses to *Piscirickettsia salmonis* infection and revealed that the gene networks involved in the apoptotic, cytoskeletal reorganization, bacterial invasion, and intracellular trafficking processes are tightly associated with disease resistance/susceptibility [29]. We are originally inspired by the previous research results in mammals and fish that the dysfunction of host immune responses to pathogen infections causes tissue hemorrhagic sepsis. In the present study, therefore, a comparative transcriptome strategy is adopted to reveal the immune gene expression profiles in two phenotypes of Chinese soft-shelled turtles with different susceptibility to *A. hydrophila* infection at different time periods (6, 24, and 72 hpi), and to clarify which immune process abnormalities may be related to the occurrence of hemorrhagic sepsis. The results depict the full picture of immune mechanisms in response to *A. hydrophila* infection, suggesting that the dysfunction of cytokine–cytokine receptor interaction, PRRs mediated signaling pathways, phagocytosis, and apoptosis may cause hemorrhagic sepsis during *A. hydrophila* infection in Chinese soft-shelled turtles. This study, for the first time, reveals the host immunity–pathogen infection interaction and molecular immunopathogenesis during *A. hydrophila* infection by comparative transcriptomes, which may contribute to the development of novel management strategies for disease control and prevention in Chinese soft-shelled turtles.

## 2. Materials and Methods

### 2.1. Experimental Animals and Bacteria Strain

Experimental turtles (*T. sinensis*) with an average weight of 16.45 ± 1.28 g were obtained from a breeding farm (111°97′ E, 28°90′ N) in Changde City, Hunan Province, China. All the turtles were acclimated in 50 L aquarium with aerated freshwater in a constant temperature laboratory room at 30 °C for two weeks before processing and were fed with a commercial diet (Kesheng Feed Stock Co., Ltd., Hangzhou, Zhejiang, China) twice a day. The animal experiments were according to the rules of the Animal Care and Use Committee of Hunan Agricultural University (Changsha, China; Approval Code: 201903297; Approval Date: 11 October 2019).

The bacteria *A. hydrophila*, isolated from the spleen of clinically diseased turtles, was provided by Professor Zhipeng Gao from the College of Animal Science and Technology, Hunan Agricultural University, China. *A. hydrophila* was cultured in Luria Bertani (LB) medium at 30 °C with the shaking at 200 rpm. After 18 h culturing, the bacteria were harvested by centrifugation, and suspended with sterile phosphate buffer saline (PBS). The bacteria concentration was adjusted to 1.39 × 10^9^ CFU/mL with PBS, which was previously proved as the medium lethal concentration for turtles during *A. hydrophila* infection.

### 2.2. Experimental Treatments, Pathological Observation, and Sampling

For the *A. hydrophila* challenge, 90 turtles were intraperitoneally injected with bacteria suspension (100 μL per turtle, designated as the treatment group). After bacteria injection, the turtles in the treatment group could be divided into two subgroups based on their pathological symptoms and behavior activities. One subgroup of turtles showed weaker ability to feed and move, with significant hemorrhagic symptoms on the body surface and swelling and congestion on viscera after anatomy, which was designated as the inactive subgroup. The other subgroup of turtles exhibited no obvious pathological symptoms, which was designated as the active subgroup. Ten turtles, injected with 100 μL of PBS, were set up as the control group.

To investigate the expressional profiles of immune-related genes during *A. hydrophila* infection in active and inactive turtles, important immune-related tissues including spleen and liver from the active subgroup turtles (*N* = 3) and inactive subgroup turtles (*N* = 3) were sampled post-injection of *A. hydrophila* at 6, 24, 72 h, respectively. Spleen and liver tissues from the control group turtles (*N* = 3) with PBS injection at 0 h were sampled as the control for the gene expression comparison with those from active subgroup turtles or from inactive subgroup turtles. Each sample was taken three times, and the sampled tissues were stored in liquid nitrogen before RNA extraction.

### 2.3. RNA Extraction, Library Preparation, and RNA Sequencing (RNA-Seq)

Total RNA from each sampled tissue was isolated by using RNeasy mini kit (QIAGEN, Germantown, MD, USA) and treated with RNase-free DNase I (QIAGEN, Germantown, MD, USA) at 37 °C for 1 h to remove residual genomic DNA. RNA quality and concentration were determined using Agilent Bioanalyser 2100 (Agilent Technologies, Santa Clara, CA, USA) and NanoDrop-1000 (NanoDrop Technologies, Wilmington, DE, USA), respectively. Five micrograms of RNA were used to construct RNA-seq library according to the instruction of Illumina mRNA-Seq Prep Kit (Illumina, San Diego, CA, USA), and the libraries were sequenced by paired-end sequencing on the Illumina HiSeq 2500 sequencing platform (Illumina, San Diego, CA, USA). The quality of RNA-Seq raw reads were assessed with FastQC (version 0.10.1; http://www.bioinformatics.bbsrc.ac.uk/projects/fastqc/ accessed on 11 March 2019), and were cleaned by removing adapter sequences, poly-N sequences, and low-quality sequences. The clean reads were then aligned to the published *T. sinensis* genome using HISAT2 [30]. In total, 291.16 GB clean data were produced, and the average of 20.4~36.3 GB clean reads were obtained from samples. About 80% of the clean reads were mapped to the reference genome by using StringTie software (Center for Computational Biology, Johns Hopkins University, Baltimore, MD, USA) [31], and the Q30 was >92.86%. The general information of RNA-Seq data is listed in Table S1.

### 2.4. Identification of the Differentially Expressed Genes (DEGs)

The relative transcript abundances in tissues (liver and spleen) from active and inactive subgroup turtles at different time periods of *A. hydrophila* infection compared to the control turtles were, respectively, estimated by using StringTie software with expectation maximization method, based on fragments per kilobase of exon per million fragments mapped (FPKM) [31]. Differential expression analysis was performed by using the DESeq R package with default parameters [32]. Benjamini and Hochberg’s approach was used to control the false discovery rate (FDR) by resulting *P*-values adjustment [33]. Genes with an adjusted *P*-value (or q-value) < 0.05 found by DEGSeq were assigned as the differentially expressed.

### 2.5. KEGG Pathway Enrichment Analysis

Significantly enriched signal transduction pathways represented by DEGs were determined using KEGG pathway enrichment analysis, compared with the whole genome background [34]. The statistical enrichment of DEGs in KEGG pathways was tested using the software KOBAS, with a *P*-value < 0.05 [34]. The significantly enriched KEGG pathways are listed in Tables S2–S5.

### 2.6. Gene Expression Validation Using Quantitative PCR (qPCR)

To validate RNA-seq data and gene expression profiles, nine DEGs were randomly selected to perform qPCR. Specific primers were designed using Primer 5 based on the coding sequences of identified genes from the turtle genome; the sequences of primers are listed in Table S6. Primer specificity was ascertained using the following steps: PCR amplification, sequencing of PCR products, and BLAST analysis in the NCBI database.

The RNA samples used for qPCR amplifications were the same as those used to construct the RNA-Seq library mentioned above. The qPCR was performed using the LightCycler^®^480 Real-Time PCR System (Roche, Basel, Switzerland) with SYBR Green I Master. The reaction mixture (10 μL) comprised 2.5 μL cDNA, 0.5 μL (10 nM) forward primer and 0.5 μL (10 nM) reverse primer, 1.5 μL PCR grade water, and 5 μL Master Mix. Each reaction was performed in triplicate under the following conditions: 95 °C for 10 min, 40 cycles of 95 °C for 15 s, 55 °C for 15 s, and 72 °C for 30 s. The relative expression level of each gene was calculated according to the 2^−△△Ct^ method [35] and normalized to the endogenous control genes of β-actin and GAPDH.

## 3. Results

### 3.1. Symptom Description of the Turtles Challenged with A. hydrophila

**Figure 1:**
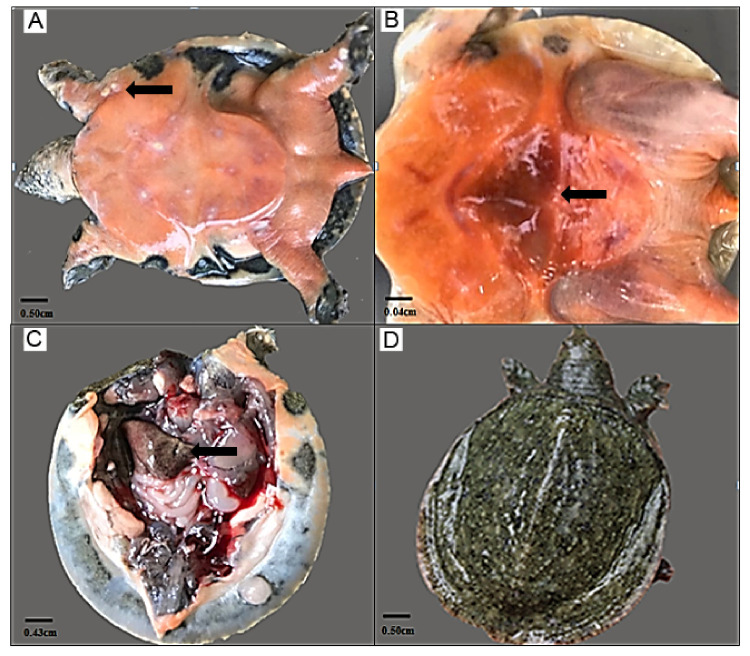
Pathological symptoms of turtles infected by *A. hydrophila*. The pathological symptoms on the body surface including (**A**) white spots near the axillae; (**B**) abdominal congestion in inactive subgroup turtles. (**C**) The pathological symptoms of viscera including liver, spleen, and intestines after anatomy of inactive turtles. (**D**) The active subgroup turtles showed no obvious pathological symptoms and were aggressive and active in feeding and moving. Black arrows indicate the location of pathological symptoms.

Ninety Chinese soft-shelled turtles were injected with *A. hydrophila* (1.39 × 10^9^ CFU/mL), and the pathological symptoms of turtles were observed. There were no obvious pathological symptoms on the body surface of turtles, and only six turtles showed behavior abnormality with slow-moving action at 6 hpi (post-injection of 6 h). At 24 hpi, the food intake and movement of turtles was overall reduced, and seven turtles died (accounting for about 8%), with the pathological symptom of abdominal congestion. At 72 hpi, 34 turtles (accounting for about 38%) died. The pathological symptoms on the body surface of diseased turtles were obvious, with white spots near the axillae, swelling, and congestion in chest and abdomen (Figure 1A,B). After anatomizing the diseased turtles, pathological symptoms of turtle viscera were easily observed (Figure 1C). The liver and spleen were swelling and congestive, with the tendency to decay, and the color of liver was yellow (Figure 1C). The intestines were filled with no food debris, and the color was white (Figure 1C). There were 13 turtles (accounting for about 14%) without any obvious pathological symptoms at 72 hpi (Figure 1D). During the whole experiment within 72 h, 10 turtles in the control group showed no obvious pathological symptoms and were aggressive and active in feeding and moving (data not shown).

According to pathological symptoms, the Chinese soft-shelled turtles challenged with *A. hydrophila* could be divided into two subgroups, including the active and inactive turtles. The liver and spleen were important immune organs in turtles, and both carried obviously pathological symptoms after *A. hydrophila* infection. Therefore, liver and spleen samples from active and inactive subgroup turtles were taken at 6, 24, and 72 hpi, respectively, and transcriptome sequencing was performed to reveal the differences of gene expression profiles between active and inactive turtles infected by *A. hydrophila*.

**Figure 2:**
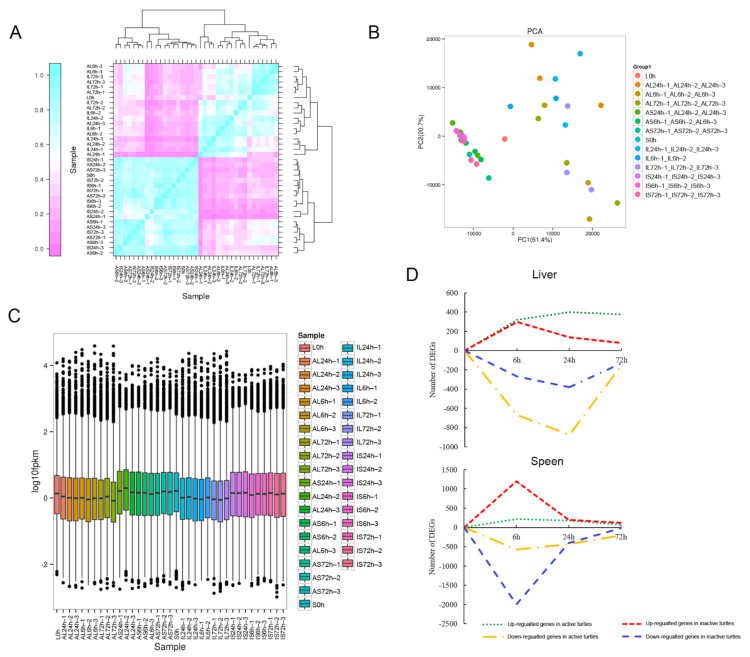
Transcriptional relationship between samples and the overall gene expression profiles. (**A**) Heatmap of correlation value (R square) of 37 libraries from liver or spleen samples. (**B**) Principal component analysis based on all of the expressed genes, showing 14 distinct groups of samples. (**C**) The dispersion degree of the gene expression level in a single liver or spleen sample. (**D**) The significantly up-regulated and down-regulated DEGs identified in livers or spleens from active and inactive turtles at 6, 24, and 72 hpi compared to the control.

Pearson’s correlation coefficients were first used to test for biologically repeated correlations between samples (Figure 2A). The generated cluster dendrogram was used to observe the overall correlation of the transcriptomes from the AL group (liver in active turtles), the IL group (liver in inactive turtles), the AS group (spleen in active turtles), and the IS group (spleen in inactive turtles) at different time periods (6, 24, and 72 hpi) (Figure 2A). Three biological replicates of liver and spleen samples from each time period and the transcriptome data both exhibited good correlation (Figure 2A). The similarity test between the three biological replicates required the use of a principal component analysis (PCA) (Figure 2B). Using the first principal component (PC1) and second principal component (PC2), a dimensionality reduction analysis was used to analyze the similarity between each replicate (Figure 2B). Figure 2B showed that biological replicates of samples overall exhibited good similarity. The generated box plot presented the dispersion degree of the gene expression level in a single liver or spleen sample, and intuitively revealed the whole gene expression level difference among all samples (Figure 2C). The results showed that the gene expression level in the spleen was overall higher than that in the liver (Figure 2C).

Totals of 4092 and 5793 DEGs were obtained in the liver and spleen transcriptomes, respectively (Figure 2D). In the AL group, 321, 401, and 378 genes were significantly up-regulated, and 671, 874, and 151 genes were significantly down-regulated at 6, 24, and 72 hpi, respectively (Figure 2D). In the IL group, the numbers of significantly up-regulated genes were 298, 138, and 79; the numbers of significantly down-regulated genes were 268, 381, and 132 at 6, 24, and 72 hpi, respectively (Figure 2D). In the AS group, 219, 176, and 65 genes were significantly up-regulated, and 576, 437, and 206 genes were significantly down-regulated at 6, 24, and 72 hpi, respectively (Figure 2D). In the IS group, the numbers of significantly up-regulated genes were 1193, 203, and 117; the numbers of significantly down-regulated genes were 1996, 401, and 204 at 6, 24, and 72 hpi, respectively (Figure 2D). The results revealed that the number of DEGs in the spleen transcriptomes was more than that in the liver transcriptomes, and the molecular response peaked at 24 hpi in liver, while it peaked at 6 hpi in spleen (Figure 2D).

### 3.2. Functional Classification of DEGs in Turtle Liver Transcriptomes by KEGG

In order to investigate the different molecular response mechanisms against *A. hydrophila* infection in livers from active and inactive turtles, the functional classification of DEGs in AL and IL group transcriptomes at different time periods (6, 24, and 72 hpi) were analyzed by KEGG enrichment analysis, and the results are summarized as follows.

#### 3.2.1. Sequential Changes of KEGG Enrichment in AL Group Turtles

**Figure 3:**
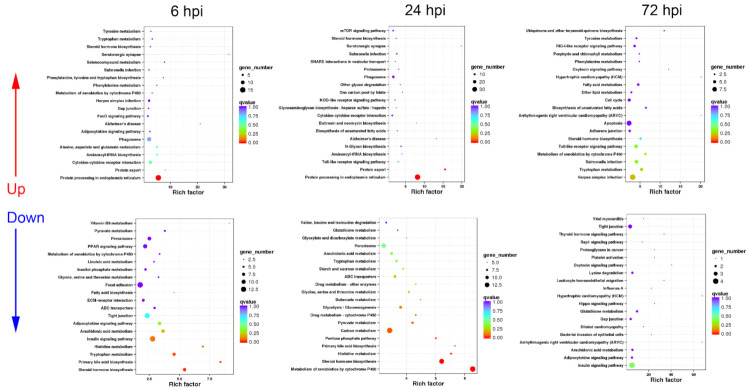
KEGG enrichment analysis in AL group turtles at different time periods. The top 20 KEGG pathways are presented here in the form of scatterplots to show the up-regulated and down-regulated DEGs enriched in livers from active subgroup turtles at 6, 12, and 72 hpi. The enrichment factor is the ratio between the DEG number and the number of all genes in a certain gene enrichment term. The sizes of the dots on these plots denote the number of DEGs, while colors correspond to the q value range.

In AL group, the up-regulated DEGs were mainly enriched in immune-related pathways including “cytokine–cytokine receptor interaction”, phagocytosis-associated processes including “phagosome”, “protein processing in endoplasmic reticulum”, and “protein export”, and pathogen infection-related pathways, while the down-regulated DEGs were intensively involved in 17 metabolism pathways and three cell adhesion-related processes at 6 hpi (Figure 3).

At 24 hpi, the majority of up-regulated DEGs were also annotated into immune-related pathways including “cytokine–cytokine receptor interaction”, “phagosome”, “protein processing in endoplasmic reticulum”, “proteasome”, and “protein export” (Figure 3). In addition, cytokine expression-mediating pathways including “toll-like receptor signaling pathway” and “NOD-like receptor signaling pathway” were significantly up-regulated (Figure 3). Similar to the response at 6 hpi, the down-regulated DEGs were mainly involved in a series of metabolism and cell adhesion pathways (Figure 3).

At 72 hpi, the up-regulated DEGs could be functionally classified into “toll-like receptor signaling pathway” and “RIG-I-like receptor signaling pathway”, pathogen infection-related pathways, “apoptosis”, and several metabolism-related pathways (Figure 3), while the down-regulated DEGs mainly participated in important metabolism-related pathways such as “insulin signaling pathway” and “adipocytokine signaling pathway” (Figure 3).

#### 3.2.2. Sequential Changes of KEGG Enrichment in IL Group Turtles

**Figure 4:**
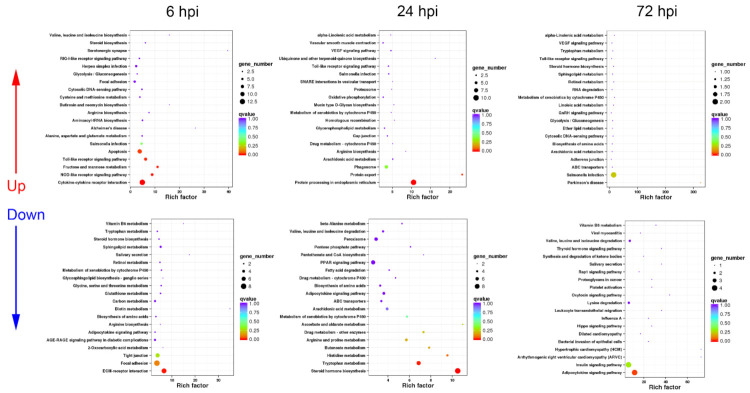
KEGG enrichment analysis in IL group turtles at different time periods. The top 20 KEGG pathways are presented here in the form of scatterplots to show the up-regulated and down-regulated DEGs enriched in livers from inactive subgroup turtles at 6, 12, and 72 hpi. The enrichment factor is the ratio between the DEG number and the number of all genes in a certain gene enrichment term. The sizes of the dots on these plots denote the number of DEGs, while colors correspond to the q value range.

Unlike the KEGG enrichment in AL group at 6 hpi, the up-regulated DEGs were mainly enriched in cytokine expression-mediating pathways including “toll-like receptor signaling pathway”, “NOD-like receptor signaling pathway”, and “RIG-I-like receptor signaling pathway” and “apoptosis”, besides “cytokine–cytokine receptor interaction”, pathogen infection-related pathways; while phagocytosis-associated processes were not listed in the top 20 up-regulated KEGG pathways in IL group at 6 hpi (Figure 4). The down-regulated DEGs were functionally annotated into cell adhesion-related pathways such as “ECM–receptor interaction” and “focal adhesion”, and energetic metabolism pathways (Figure 4).

At 24 hpi, the term of “cytokine–cytokine receptor interaction” was not listed in the top 20 up-regulated KEGG pathways, while the up-regulated DEGs were mainly involved in phagocytosis-associated processes such as “phagosome”, “protein processing in endoplasmic reticulum”, “proteasome”, and “protein export” (Figure 4). Additionally, “toll-like receptor signaling pathway” was significantly up-regulated (Figure 4). For the down-regulated DEGs, most of them were associated with hormone synthesis and amino acid metabolism (Figure 4).

At 72 hpi, immune-related terms including “toll-like receptor signaling pathway” and “cytosolic DNA-sensing pathway” were listed in the top 20 up-regulated KEGG pathways (Figure 4). The down-regulated DEGs were mainly enriched in energetic metabolism pathways such as “insulin signaling pathway” and “adipocytokine signaling pathway” (Figure 4).

#### 3.2.3. Expression Difference Analysis of Cytokine, Phagocytosis, and Apoptosis-Related Genes between AL and IL Group Turtles

The KEGG pathways related to immune processes including cytokine–cytokine receptor interaction, phagocytosis, and apoptosis enriched in AL and IL group turtles challenged with *A. hydrophila* were quite different (Table 1, Table 2 and Table 3). Therefore, the fold changes of differentially expressed cytokine, phagocytosis, and apoptosis-related genes were further analyzed.

The results showed that the sharpest response of cytokines occurred at 24 hpi in the AL group, with the up-regulation of two cytokine and two cytokine receptor genes (Table 1). Among three cytokine expression-mediating pathways, only the response of toll-like receptor signaling pathway was relatively intense, with five up-regulated genes, also at 24 hpi in the AL group (Table 1), while in IL group, the sharpest response of cytokines occurred at 6 hpi, with the up-regulation of five cytokine and two cytokine receptor genes, and few cytokines were differentially expressed at 24 or 72 hpi (Table 1). Moreover, the up-regulation of differentially expressed cytokine genes at 6 hpi in IL group was overall higher than those in AL group at any time periods (Table 1). Nevertheless, IL10, an anti-inflammatory cytokine, was not differentially expressed at any time periods in the IL group, while it was up-regulated at all the time periods in the AL group (Table 1). In addition, the up-regulations of both NOD-like receptor signaling pathway and RIG-I-like receptor signaling pathway genes were intense in the IL group at 6 hpi (Table 1).

Notably, the sharpest response of phagocytosis-related gene occurred at 6 hpi in the AL group, with the up-regulation of 10 DEGs, and lasted until 24 hpi (Table 2). While in the IL group, at 24 hpi, the phagocytosis activity seemed to just start, with five up-regulated phagocytosis-related DEGs (Table 2). In addition, the most intense response of apoptosis-related genes occurred at 6 hpi in the IL group, with the up-regulation of six DEGs, while in the AL group, the apoptosis seemed to just start at 24 hpi with the up-regulation of five DEGs (Table 3).

### 3.3. Functional Classification of DEGs in Turtle Spleen Transcriptomes by KEGG

The functional classification of DEGs in AS and IS transcriptomes at different time periods were also analyzed by KEGG enrichment to further reveal the different immune response mechanisms against *A. hydrophila* infection in spleens between active and inactive turtles. The results are summarized as follows.

#### 3.3.1. Sequential Changes of KEGG Enrichment in AS Group Turtles

**Figure 5:**
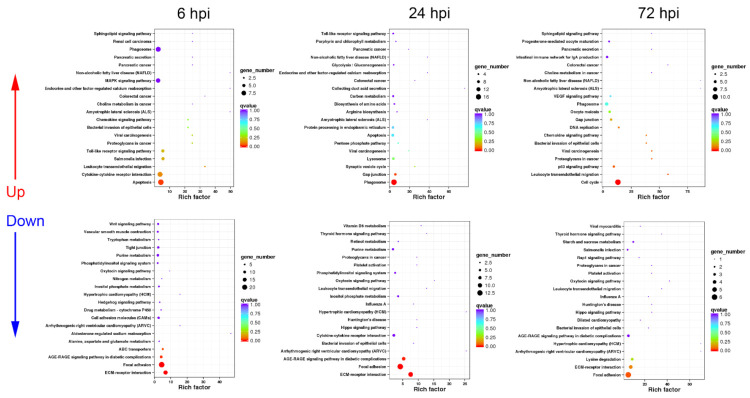
KEGG enrichment analysis in AS group turtles at different time periods. The top 20 KEGG pathways are presented here in the form of scatterplots to show the up-regulated and down-regulated DEGs enriched in spleens from active subgroup turtles at 6, 12, and 72 hpi. The enrichment factor is the ratio between the DEG number and the number of all genes in a certain gene enrichment term. The sizes of the dots on these plots denote the number of DEGs, while colors correspond to the q value range.

In the AS group, the up-regulated DEGs were functionally associated with immune processes including “cytokine–cytokine receptor interaction”, “chemokine signaling pathway”, “phagosome”, “apoptosis”, “leukocyte transendothelial migration”, “toll-like receptor signaling pathway”, “MAPK signaling pathway”, and pathogen infection-related pathways at 6 hpi (Figure 5), while the down-regulated DEGs were involved in cell adhesion and metabolism pathways at 6 hpi (Figure 5).

At 24 hpi, the up-regulated DEGs were mainly annotated into phagocytosis-related pathways such as “phagosome”, “protein processing in endoplasmic reticulum”, “synaptic vesicle cycle”, and “lysosome”. In addition, “toll-like receptor signaling pathway” was significantly up-regulated (Figure 5). Unlike the response at 6 hpi, the down-regulated DEGs at 24 hpi could be enriched in pathways including “cytokine–cytokine receptor interaction” and “bacterial invasion of epithelial cells”, besides a series of metabolism and cell adhesion pathways (Figure 5).

At 72 hpi, the up-regulated DEGs were functionally classified into apoptosis-related pathways, including “p53 signaling pathway” and “cell cycle”, as well as immune defense processes such as “phagosome”, “chemokine signaling pathway”, and “leukocyte transendothelial migration” (Figure 5), while the down-regulated DEGs mainly participated in important metabolism and cell adhesion pathways (Figure 5).

#### 3.3.2. Sequential Changes of KEGG Enrichment in IS Group Turtles

**Figure 6:**
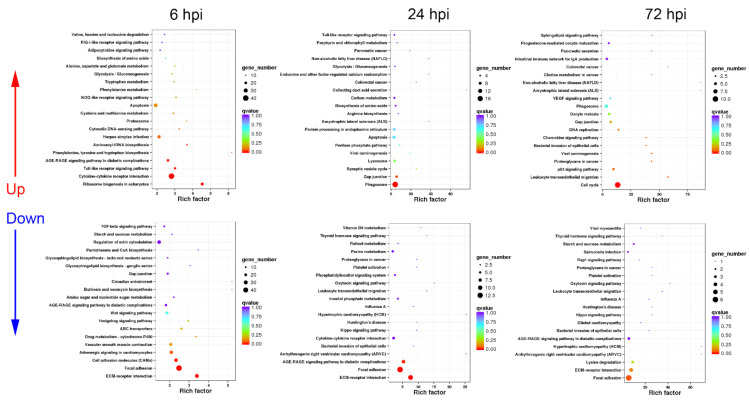
KEGG enrichment analysis in IS group turtles at different time periods. The top 20 KEGG pathways are presented here in the form of scatterplots to show the up-regulated and down-regulated DEGs enriched in spleens from inactive subgroup turtles at 6, 12, and 72 hpi. The enrichment factor is the ratio between the DEG number and the number of all genes in a certain gene enrichment term. The sizes of the dots on these plots denote the number of DEGs, while colors correspond to the q value range.

The strongest immune response of spleen occurred at 6 hpi in the IS group, with 1193 up-regulated and 1996 down-regulated DEGs. Most up-regulated DEGs were enriched in immune-related pathways including “cytokine–cytokine receptor interaction”, “toll-like receptor signaling pathway”, “NOD-like receptor signaling pathway”, and “RIG-I-like receptor signaling pathway”, as well as apoptosis-associated processes (Figure 6), while the down-regulated DEGs were functionally annotated into cell adhesion-related pathways such as “ECM–receptor interaction” and “focal adhesion”, and metabolism pathways (Figure 6).

At 24 hpi, the up-regulated DEGs could be related to phagocytosis such as “phagosome” and “lysosome” (Figure 6). Additionally, “toll-like receptor signaling pathway” was listed in the top 20 up-regulated KEGG pathways (Figure 6). For the down-regulated DEGs, most of them were associated with cell adhesion, hormone synthesis, and amino acid metabolism (Figure 6).

Similar to the response at 72 hpi in the AS group, in the IS group, the up-regulated DEGs mainly participated in apoptosis-related pathways including “p53 signaling pathway” and “cell cycle”, as well as “phagosome” and “chemokine signaling pathway” at 72 hpi (Figure 6), while the down-regulated DEGs were enriched in a series of hormone synthesis, amino acid metabolism, and cell adhesion pathways (Figure 6).

#### 3.3.3. Expression Difference Analysis of Cytokine, Phagocytosis, and Apoptosis-Related Genes between AS and IS Group Turtles

The fold changes of differentially expressed cytokine, phagocytosis, and apoptosis-related genes were also analyzed in AS and IS group turtles (Table 4, Table 5 and Table 6). The results showed that in both AS and IS groups, the sharpest response of cytokines occurred at 6 hpi, while the number of differentially expressed cytokine and cytokine receptor genes in the IS group were overwhelmingly more than that in the AL group (30 up-regulated genes in the IS group vs. seven up-regulated genes in the AS group) (Table 4). Noteworthily, the overall expression of cytokines dramatically decreased at 24 hpi and 72 hpi in the IS group, while gradually decreased in the AS group, and the anti-inflammatory cytokine IL10 was up-regulated at all the time periods in the AS group (Table 4). Consistent with the response of cytokines, the expression of toll-like receptor signaling pathway genes were also relatively intense at 6 hpi, but only with three up-regulated genes in the AS group (Table 4), while in the IS group at 6 hpi, the expressions of three cytokine expression-mediating pathway genes, including toll-like receptor signaling pathway, NOD-like receptor signaling pathway, and RIG-I-like receptor signaling pathway, were overall up-regulated, with 18 up-regulated genes (Table 4).

The intense response of phagocytosis started at 6 hpi in the AS group, with the up-regulation of seven DEGs, and lasted until 24 hpi (six up-regulated DEGs) (Table 5), while in the IS group at 6 hpi, the phagocytosis activity seemed to be inhibited, with 10 down-regulated DEGs, and just started at 24 hpi, with 11 up-regulated phagocytosis-related DEGs (Table 5). Nevertheless, in both the AS and IS groups, the sharpest response of apoptosis-related gene expression occurred at 6 hpi, while the number of up-regulated apoptosis-related genes in the IS group (18 DEGs) was significantly more than that in the AL group (four DEGs) (Table 6).

### 3.4. Validation of DEGs by qPCR

**Figure 7:**
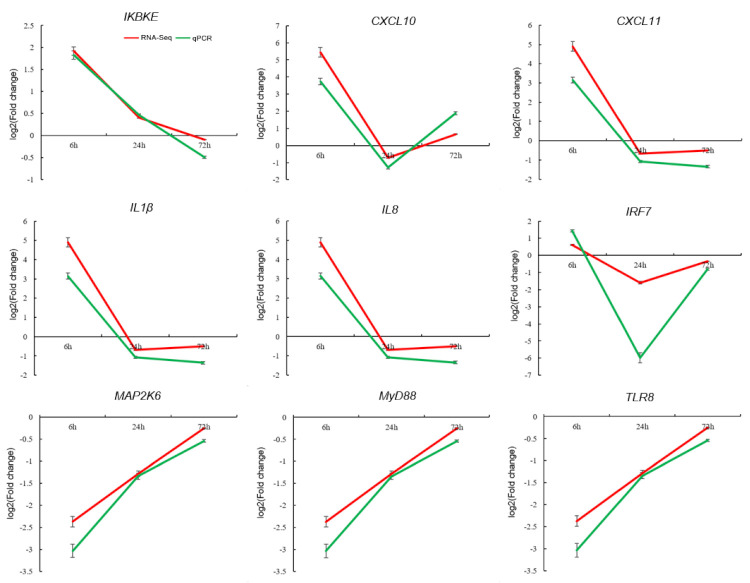
Validation of RNA-Seq results by qPCR. Nine DEGs are randomly selected, and the expressions of genes at different time periods are examined relative to the endogenous control genes (β-actin and GAPDH). The relative expression values are transformed into the log_2_ (fold change) form. The results are shown as the mean ± SEM of liver and spleen tissues derived from 3 individual turtles.

A total of nine DEGs were randomly selected to perform qPCR to validate RNA-Seq data and gene expression profiles (Figure 7). PCR products with expected sizes were successfully amplified with all the nine specific primer pairs, indicating their availabilities for DEG validation (data not shown). The different amplification efficiencies of the nine DEGs between the experimental and control groups were transformed by log_2_ (fold change) to compare with the results of RNA-Seq. The results showed that the expression patterns of these genes determined by qPCR were similar to those acquired through RNA-Seq (Figure 7), which confirmed the reliability of the RNA-Seq data. Therefore, the immune-related genes isolated in this study could be useful references for future studies on the molecular mechanisms of Chinese soft-shelled turtles during *A. hydrophila* infection.

## 4. Discussion

For the nutriment and medical values, Chinese soft-shelled turtle *T. sinensis* has been developed into the largest cultured turtle species in East Asia, especially in China and Japan. Serious infectious diseases caused by pathogens including bacteria and viruses is threatening the aquaculture of turtles [3]; in particular, the hemorrhagic sepsis caused by *A. hydrophila*, with more than 15 kinds of diseases, is the most common and troublesome in turtle disease cases [36,37]. Previous studies have reported in mammals and fish that abnormal immune responses to pathogenic infections, such as excessive activation of immune cells and dysfunction of immune responses, can lead the immune system to attack self-uninfected cells, causing systemic inflammation, tissue hemorrhagic sepsis, and even death [38,39]. However, the research on the immune response mechanisms is limited, and the molecular pathology of turtles infected by *A. hydrophila* remains unclear, which greatly hinders the strategy innovations for disease prevention and control in Chinese soft-shelled turtles.

The susceptible and resistant individuals in the natural population have offered excellent materials to study the molecular pathology or molecular basis of resistance for pathogenic diseases in many animal species [40,41]. In livestock and poultry animals, for example, comparative transcriptomes were analyzed to reveal the molecular mechanism differences in response to *Mycoplasma hyopneumoniae* infection in two pig breeds [42]. These two breeds share DEGs that are involved in immune relevant pathways, including cytokine–cytokine receptor interaction pathway, PI3K-Akt signaling pathway, and chemokine signaling pathway [42]. The study demonstrates that more chemokines and interleukins are specifically and significantly up-regulated, which can enhance the immune responses and reduce the susceptibility to *M. hyopneumoniae* infection in resistant pig breed [42]. When cytokine gene expressions are compared between chicken line 6.3 (Marek’s disease-resistant chicken) and line 7.2 (Marek’s disease-susceptible chicken) in a transcriptome analysis, among the identified 53 cytokines and 96 cytokine receptors, 15 cytokines and 29 cytokine receptors highly expressed in line 6.3 were detected [43]. In aquaculture fish species, critical cytokines including, IL8 and TNFα, are significantly up-regulated in resistant channel catfish (*Ictalurus punctatus*), while susceptible catfish show high expression levels of IL17 in response to *Flavobacterium columnare* infection [44]. The gene networks involved in the apoptotic process are also associated with disease resistance/susceptibility to *Piscirickettsia salmonis* in Atlantic salmon [29]. This evidence indicates that the expression of immune-relevant genes interrelates with disease resistance/susceptibility to pathogenic diseases in animal hosts. In the present study, two phenotypes of Chinese soft-shelled turtles were found after *A. hydrophila* infection. One group of turtles were active in feeding and moving, with no obvious pathological symptoms, which were considered as the resistant turtles, while the other group of turtles showed obviously pathological symptoms, with swelling and congestion in liver and spleen after *A. hydrophila* infection and the reduction of food intake and movement, which are regarded as susceptible turtles. Comparative liver and spleen transcriptomes from these two groups of turtles at different time periods (6, 24, and 72 hpi) were further analyzed to reveal the molecular basis of resistance/susceptibility for turtles infected by *A. hydrophila*. The results indicate that the expression of cytokine, apoptosis-, and phagocytosis-related genes in both liver and spleen of the inactive turtles is significantly distinct from those in the active turtles analyzed by KEGG pathway enrichment. Therefore, we infer that these gene expression differences may be related to the molecular pathology or resistant basis to *A. hydrophila* infection in Chinese soft-shelled turtles.

Cytokines are a class of low-molecular-weight-secreted proteins that can transduce signals between cells and exert immune regulation and effector functions [45]. They play important roles in the immune system by regulating the intensity and duration of immune responses [46]. During pathogen infection, cytokines produced by immune cells trigger an inflammatory response, which is essential for the early elimination of pathogens [47]. However, the lasting or excessive production and release of cytokines may initiate the “cytokine storm”, which often leads to various diseases, including hemorrhagic septicemia and even the failure of key organs or death for animal hosts [48]. It has been reported in many aquaculture fish species that abnormal expression of cytokines is linked to serious hemorrhagic septicemia. For example, hemorrhagic septicemia of mandarin fish (*Siniperca chuatsi*) is mainly caused by *A. hydrophila* infection [49]. Histopathological analysis reveals that inflammation, vacuolization, and extensive necrosis exist in the gill, liver, spleen, and head kidney of the diseased mandarin fish [49]. The mRNA expression levels of pro-inflammatory cytokines including TNFα, CCL3, and IL8 are sharply up-regulated in spleen and head kidney of mandarin fish post-*A. hydrophila* infection [49]. Coincidentally, Chinese perch infected with *A. hydrophila* also shows significantly high mRNA expression levels of pro-inflammatory cytokines, such as TNFα, and IL1β, compared to healthy fish [50]. In tambaqui (*Colossoma macropomum*), IL1β and complement component 4 are intensely up-regulated post-*A. hydrophila* infection [51]. With transcriptome analysis, the KEGG pathways associated with disease and immune responses, such as the cytokine–cytokine receptor interaction, complement and coagulation cascades, and inflammatory bowel disease, are also enriched in *Leiocassis longirostris* with *A. hydrophila* infection [52]. This evidence has suggested the involvement of pro-inflammatory cytokines in pathogenesis of hemorrhagic septicemia caused by *A. hydrophila* infection in fish. In the present study, the up-regulated number of cytokine and cytokine receptor genes are far more, and their up-regulations are more intense in inactive turtles than those in the active turtles. Especially in the spleen of inactive turtles, the significant up-regulations of 12 pro-inflammatory cytokines, including IL1β, IL6, IL8, CCL20, CXCL10, CXCL11, CXCL13, CXCL14, CX3CL1, TNFSF8, TNFSF15, TNFSF18, and 17 cytokine receptors, were identified at 6 hpi. The excessive expression of pro-inflammatory cytokines and their receptors have been confirmed to bring about uncontrolled inflammation, and lead to the pathological changes in tissues or key organs, and systemic hemorrhagic sepsis [48]. Since the high expression of pro-inflammatory cytokines, it is reasonable that the spleen and liver of inactive turtles exhibits the symptoms of hemorrhagic sepsis after *A. hydrophila* infection. In addition, it is worth noting that in both liver and spleen of the active turtles, IL10 is significantly up-regulated at all the tested time periods (6, 24, and 72 hpi). IL10 is well known as an important anti-inflammatory cytokine, which can prevent excessive tissue damage caused by bacterial and viral infections as well as pro-inflammatory responses [53]. Especially in the late phase of pathogen infection, IL10 serves the role in controlling the development of inflammatory diseases [54,55]. These above results collectively hint that the excessive expression of a large number of pro-inflammatory cytokines (“cytokine storm”) triggers an imbalanced immune response, which should partly explain the molecular pathology of hemorrhagic sepsis in the liver and spleen of inactive turtles, while the lasting up-regulation of IL10 may be critical for maintaining the immune homeostasis in the active turtles during *A. hydrophila* infection.

In fact, the expression of cytokines is delicately induced and regulated, where signal pathways mediated by PRRs such as TLRs, NLRs, and RLRs undertake the indispensable roles [56]. The innate immune cells utilize PRRs to recognize the invading microorganisms, and trigger downstream immune-related signal cascades [56]. Although the downstream signaling pathways mediated by TLRs, NLRs, and RLRs are different, they all induce the production of specific cytokines. For example, the activation of TLR signaling pathway usually induces the expression of pro-inflammatory cytokines including TNFs, interleukins such as IL1β, IL6, IL8, and IL12, chemokines including CCL3 and CCL5, and interferon genes [57]. It is well known that the activation of RLR signaling pathway initiates interferon production to resist virus, and it can also induce the expression of TNFs, IL8, IL12, and other pro-inflammatory cytokines depending on NF-κB phosphorylation [58], while the activation of NLR signaling pathway is only associated with the induction of pro-inflammatory cytokines such as TNFs, IL1β, IL6, IL8, IL12, CCL3, and CCL5 [59]. In the present study, we found that in both the active and inactive turtles, the TLR-, NLR-, and RLR-mediated signaling pathways exhibited different degrees of activation along with the up-regulation of cytokines after *A. hydrophila* challenge. The difference is that in liver and spleen of the active turtles, only the activation of the TLR signaling pathway is relatively intense when the cytokine expression is peaking at 24 hpi, while in liver and spleen of the inactive turtles, all the TLR, NLR, and RLR signaling pathways are significantly activated when the cytokine expression is peaking at 6 hpi. It has been reported in mammals and several fish species that immoderate activation of the PRRs-mediated signaling pathways causes excessive expression of pro-inflammatory cytokines, leading to the dysfunction of immune regulation and inflammatory disease [56,60]. Similarly, extensive activation of PRRs-mediated signaling pathways may be the key reason for the excessive expression of pro-inflammatory cytokines, which can explain the molecular pathology of hemorrhagic sepsis in inactive turtles after *A. hydrophila* infection.

Apoptosis is defined as programmed cell death, involved in many physiological processes including homeostasis maintenance, and developments of tissue and organ [61]. Conceptually, cell death appears to protect against most acute bacterial pathogens that infect hosts and, in many cases, even more successfully restricts nonpathogenic or opportunistic bacteria [62]. Therefore, apoptosis is considered as an intrinsic immune defense mechanism in response to microbial infections, and the apoptosis of infected cells is an effective way to eliminate pathogenic niches and prevent their further spreading [63]. It has been reported that bacterial infection sensed by PRRs induces NF-κB-dependent inflammatory cytokines, including those of the TNFs and ILs, which further promote inflammatory signaling through death receptors and induce apoptosis [64]. In Japanese flounder, cell apoptosis, along with the up-regulation of NLRP3, ASC, caspase-1, IL1β, and IL18, in the macrophages has been observed after *Edwardsiella tarda* infection [65]. In addition, the apoptosis of erythrocytes can be induced by *A. hydrophila* infection in grass carp, along with the up-regulation of CCL4, CCL11, CCL20, IL4, and IL12 [66]. These studies indicate that the inflammation caused by bacterial infection is often accompanied by cell apoptosis in hosts. In the present study, we found that in active turtles, only several apoptosis-related genes were significantly up-regulated at 6 hpi in both liver and spleen, and their expressions gradually decreased at 24 and 48 hpi, while in inactive turtles, the up-regulation of a large number of apoptosis-related genes, as well as inflammatory cytokines, including TNFs, ILs, and chemokines, were observed at 6 hpi, and the up-regulation of apoptosis-related genes can last to 72 hpi in both liver and spleen. These results confirm that the up-regulation of apoptosis-related genes in the inactive turtles is more intense and lasting than that in the active turtles. Since exuberant cell apoptosis often accompanies tissue damage and causes the pathological changes in parenchymal organs [67], excessive apoptosis-related gene expression may also be involved in the molecular pathology of hemorrhagic sepsis in the liver and spleen of turtles after *A. hydrophila* infection.

Phagocytosis constitutes an important immune response of immunocytes as the first line of defense to recognize and engulf foreign particles or self-apoptotic cells, followed by the digestion and clearance [68]. It is a primitive conserved innate immune defense mechanism for all metazoans, including vertebrates and invertebrates [69]. Macrophages, neutrophils, and dendritic cells are professional phagocytes that are able to phagocytose large foreign particles (with the diameter of >0.5 μm) such as bacteria [70]. Effective phagocytosis requires two components: particle internalization and phagosome maturation [71]. After the bacteria are recognized by the phagocytes, they undergo endocytosis to form the phagosome in the phagocytes [71]. The nascent or early phagosome has no killing activity, and they must transform into the mature phagosome to obtain the bactericidal properties [71]. The mature phagosome further fuses with the lysosome to form the phagolysosome where there are various bactericidal substances such as reactive oxygen species, and hydrolytic enzymes, including protease, polysaccharase, nuclease, and lipase, that can kill and digest the invading bacteria [71]. After digestion, most of the bacterial residues are discharged outside the host phagocytes, and part of the bacterial degradation products are presented onto the surface of antigen-presenting cells by MHC molecules, which promotes adaptive immune responses [72]. In the present study, we observed the expression differences of phagocytosis-related genes that are involved in the processes including internalization and formation of the phagosomes, early/mature phagosome, phagolysosome, activation of NADPH oxidase, and antigen presentation between the active and inactive turtles in response to *A. hydrophila* infection. The results indicate that in active turtles, most of the phagocytosis-related genes are significantly up-regulated at 6 hpi and the up-regulation could last until 24 and 72 hpi in both liver and spleen, while in the inactive turtles, only several phagocytosis-related genes are significantly up-regulated in the liver, and even the majority of phagocytosis-related genes are significantly down-regulated in the spleen at 6 hpi; up to 24 hpi, most of the phagocytosis-related genes are significantly up-regulated in the liver and spleen. At 72 hpi, only a small part of the phagocytosis-related genes is up-regulated. Overall, the activity of phagocytosis in the inactive turtles starts later than that in the active turtles. These results suggest that the lag of phagocytosis can lead to the inability to clear bacteria, which also may be one of the important reasons for the persistent inflammation caused by *A. hydrophila* proliferation in the inactive turtles.

## 5. Conclusions

**Figure 8:**
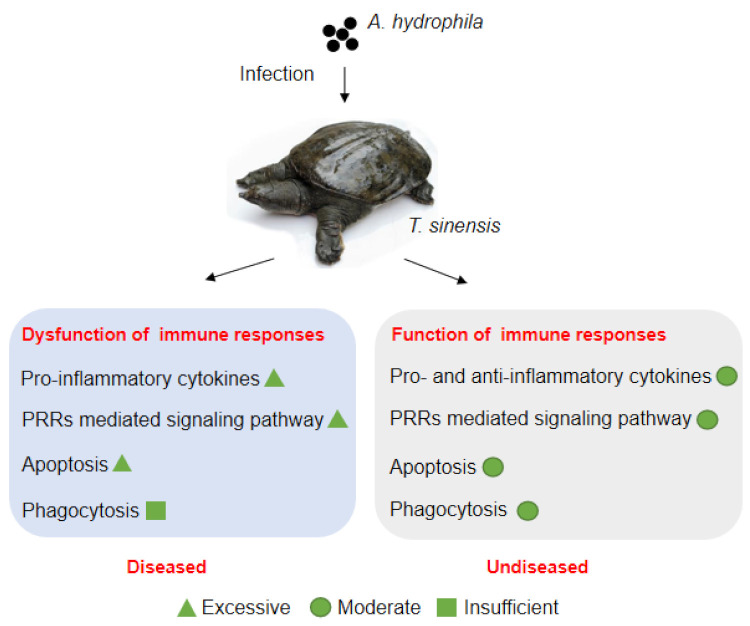
The suspected molecular immunopathogenesis of hemorrhagic sepsis caused by *A. hydrophila* infection in Chinese soft-shelled turtles.

In summary, the molecule immune responses of turtles infected by *A. hydrophila* was analyzed, for the first time, by comparative transcriptomes from two group of turtles with different susceptibility to *A. hydrophila* infection. The gene expression profiles indicate that the dysfunction of immune responses, including excessive activation of pro-inflammatory cytokines, PRRs-mediated signaling pathway, and apoptosis, and insufficient phagocytosis activity may contribute to the molecular pathology of hemorrhagic sepsis in liver and spleen of turtles during *A. hydrophila* infection (Figure 8). Although there was a lack of further functional verification for the suspected genes, the data of comparative transcriptomes in this study will provide useful information for future studies on the molecular immunopathogenesis after *A. hydrophila* infection or genetic improvements against hemorrhagic sepsis in Chinese soft-shelled turtles.

## Figures and Tables

**Table 1:** Fold changes of differentially expressed cytokine and cytokine receptor, and cytokine expression mediating pathway genes in the AL group and the IL group compared to the control.

Categories/ Gene Name	Description	Log_2_ (Fold Changes)					
		AL Group			IL Group		
		6 Hpi	24 Hpi	72 Hpi	6 Hpi	24 Hpi	72 Hpi
Interleukins and interleukin receptors							
*IL1β*	interleukin 1 beta		2.18		4.82		
*IL8*	interleukin 8				3.55		
*IL10*	interleukin 10	6.08	4.41	2.31			
*IL1R1*	interleukin 1 receptor type I		1.66				
*IL1R2*	interleukin 1 receptor type II	5.88	3.16		7.06		
*IL5RA*	interleukin 5 receptor alpha		−2.57			−2.40	−3.01
*IL18R1*	interleukin 18 receptor 1		−5.30				
Chemokines and chemokine receptors							
*CCL5*	C-C motif chemokine 5				4.71		
*CCL20*	C-C motif chemokine 20	9.34	7.63		12.18		
*CX3CL1*	C-X3-C motif chemokine 1		−4.91		3.28		
*CCR5*	C-C chemokine receptor type 5	3.02					
TNF family members and TNF receptors							
*TNFSF10*	tumor necrosis factor ligand superfamily member 10	−2.56	−2.08				
*TNFSF15*	tumor necrosis factor ligand superfamily member 15						−2.40
*SF6B*	tumor necrosis factor receptor superfamily member 6B						−2.57
*SF12A*	tumor necrosis factor receptor superfamily member 12A	4.49			4.60		
Toll-like receptor (TLR) signaling pathway							
*TLR2*	toll-like receptor 2	4.56	2.80				
*TLR5*	toll-like receptor 5		3.61	1.51			2.89
*TRIF*	toll-like receptor adapter molecule 1	−3.15	−2.87	−1.99		−3.40	−3.42
*TAB1*	TAK1-binding protein 1		1.77				
*PI3K*	phosphoinositide-3-kinase		2.48		2.96		
*MAP2K1*	mitogen-activated protein kinase kinase 1		2.13				
*MAP2K6*	mitogen-activated protein kinase kinase 6	−2.04	-2.63			−2.59	
*AP-1*	proto-oncogene protein c-fos			1.43			
*STAT1*	signal transducer and activator of transcription 1			1.49			
RIG-I-like receptor (RLR) signaling pathway							
*RIG-I*	retinoic acid inducible gene I			2.38			
*LGP2*	laboratory of genetics and physiology 2			2.21			
*MDA5*	melanoma differentiation-associated gene 5	2.43					
*TRAF3*	TNF receptor-associated factor 3				5.07	3.54	
*IRF7*	interferon regulatory factor 7		−1.83		2.86		
*DDX3X*	ATP-dependent RNA helicase DDX3X		−1.86	−1.69		−2.08	−2.02
NOD-like receptor (NLR) signaling pathway							
*ASC*	apoptosis-associated speck-like protein containing a CARD	1.99					
* RIPK2 *	receptor-interacting serine/threonine-protein kinase 2				2.47		
* cIAP *	baculoviral IAP repeat-containing protein 2/3				2.50		
* TNFAIP3 *	tumor necrosis factor alpha-induced protein 3			1.56	3.75		

**Note:** This table and the following tables only show the DEGs with the values of log_2_ (fold change) with FDR < 0.05.

**Table 2:** Fold changes of differentially expressed phagocytosis-related genes in the AL group and the IL group compared to the control.

Categories/ Gene Name	Description	Log_2_ (Fold Changes)					
		AL Group			IL Group		
		6 Hpi	24 Hpi	72 Hpi	6 Hpi	24 Hpi	72 Hpi
Internalization and formation of the phagosomes							
*TLR2*	toll-like receptor 2	4.56	2.80				
*MR*	mannose receptor		1.87	1.07		−2.18	
*iC3b*	the fragment of complement component 3	4.38		2.79			
*Collectin*	C-type lectin	4.81	3.69	1.47	−2.63		
*F-actin*	actin beta/gamma 1	5.57	5.31	4.39	5.09	5.68	4.64
Early phagosome							
*Rab5*	ras-related protein Rab-5B	2.28					
*vATPase*	V-type H^+^-transporting ATPase	2.33	2.05		3.85	−2.08	
*CALR*	calreticulin		4.05				
Mature phagosome							
*TUBA*	tubulin alpha	2.26					
*TUBB*	tubulin beta	4.63	3.25	1.42		2.72	
*vATPase*	V-type H^+^-transporting ATPase	2.33	2.05		3.85	−2.08	
Phagolysosome							
*sec61*	protein transport protein SEC61 subunit beta	2.72	3.26			2.95	
*vATPase*	V-type H^+^-transporting ATPase	2.33	2.05		3.85	−2.08	
Activation of NADPH oxidase							
*p40phox*	neutrophil cytosolic factor 4	3.50					
*p47phox*	neutrophil cytosolic factor 1		2.79			2.42	
*gp91*	NADPH oxidase 1			1.59			
Antigen presentation							
*MHC II*	MHC class II antigen		−2.21				
*sec22*	vesicle transport protein SEC22		4.21			3.54	

**Table 3:** Fold changes of up-regulated apoptosis-related genes in the AL group and the IL group compared to the control.

Gene Name	Description	Log_2_ (Fold Changes)					
		AL Group			IL Group		
		6 Hpi	24 Hpi	72 Hpi	6 Hpi	24 Hpi	72 Hpi
*p53*	tumor protein p53	1.94					
*IP3R*	inositol 1,4,5-triphosphate receptor type 3	1.35	1.56		1.92		
*Perforin*	perforin 1		3.12	2.57			
*PI3K*	phosphoinositide-3-kinase		2.48		2.96		
*MERK2*	mitogen-activated protein kinase kinase 2		2.13				
*PERK*	protein kinase RNA (PKR)-like ER kinase		1.85	2.30			
*Cathepsin*	cathepsin B				5.11	4.27	
*NOXA*	phorbol-12-myristate-13-acetate-induced protein 1			1.62			
*AP1*	proto-oncogene protein c-fos			1.43			
*GZMB*	granzyme B				3.87		
*IL3R*	cytokine receptor common subunit beta				3.43		
*A1*	hematopoietic Bcl-2-related protein A1				4.59		

**Table 4:** Fold changes of differentially expressed cytokine and cytokine receptor, and cytokine expression mediating pathway genes in the AS group and the IS group compared to the control.

Categories/ Gene Name	Description	Log_2_ (Fold Changes)					
		AS Group			IS Group		
		6 Hpi	24 Hpi	72 Hpi	6 Hpi	24 Hpi	72 Hpi
Interleukins and interleukin receptors							
*IL1β*	interleukin 1 beta				2.46		
*IL6*	interleukin 6				5.20		
*IL7*	interleukin 7				−2.79		
*IL8*	interleukin 8	3.27	2.43		2.41		
*IL10*	interleukin 10	6.82	5.69	1.21	7.30		
*IL1R2*	interleukin 1 receptor type II	4.13			3.81		
*IL1RAP*	interleukin 1 receptor accessory protein				2.24		
*IL3RB*	cytokine receptor common subunit beta				3.56		
*IL4R*	interleukin 4 receptor				1.18		
*IL5RA*	interleukin 5 receptor alpha	−1.95	−3.81			−2.85	
*IL5RB*	interleukin 5 receptor alpha				3.56		
*IL8RB*	interleukin 8 receptor	3.07			2.32		
*IL12RB1*	interleukin 12 receptor beta-1				2.60		
*IL12RB2*	interleukin 12 receptor beta-1				3.20		
*IL15RA*	interleukin 15 receptor alpha				3.68		
*IL21R*	interleukin 21 receptor				1.98		
*IL22RA2*	interleukin 22 receptor alpha 2	5.21			4.77	4.39	
Chemokines and chemokine receptors							
*CCL20*	C-C motif chemokine 20	6.33			6.72		2.56
*CXCL10*	C-X-C motif chemokine 10				5.36		
*CXCL11*	C-X-C motif chemokine 11				4.32		
*CXCL12*	C-X-C motif chemokine 12				−2.00		
*CXCL13*	C-X-C motif chemokine 13	3.47			2.41		
*CXCL14*	C-X-C motif chemokine 14				1.81		2.07
*CX3CL1*	C-X3-C motif chemokine 1		−3.70		3.90		
*CCR5*	C-C chemokine receptor type 5				1.26		
*CXCR4*	C-X-C chemokine receptor type 4					−1.75	
*XCR1*	XC chemokine receptor 1				−4.81		
TNF family members and TNF receptors							
*TNFSF8*	tumor necrosis factor ligand superfamily member 8				3.06		
*TNFSF10*	tumor necrosis factor ligand superfamily member 10		−2.86		−4.23	−3.17	
*TNFSF12*	tumor necrosis factor ligand superfamily member 12				−1.99		
*TNFSF15*	tumor necrosis factor ligand superfamily member 15				3.09		
*TNFSF18*	tumor necrosis factor ligand superfamily member 18				4.56		
*EDA*	ectodysplasin-A				−2.93		
*SF6B*	tumor necrosis factor receptor superfamily member 6B				7.29		
*SF9*	tumor necrosis factor receptor superfamily member 9				3.26		
*SF12A*	tumor necrosis factor receptor superfamily member 12A		6.32		7.32	7.37	
*SF13B*	tumor necrosis factor receptor superfamily member 13B			2.33			3.08
*SF19L*	tumor necrosis factor receptor superfamily member 19-like				5.98		
*FAS*	tumor necrosis factor receptor superfamily member 6				2.34		
*NGFR*	tumor necrosis factor receptor superfamily member 16				−3.24		
*EDAR*	ectodysplasin-A receptor				−4.72		
Toll-like receptor (TLR) signaling pathway							
*TLR4*	toll-like receptor 4				2.30	1.71	
*TLR5*	toll-like receptor 5	2.77	1.82		3.00		
*MyD88*	myeloid differentiation factor 88				1.08		
*TRIF*	toll-like receptor adapter molecule 1				1.96		
*Rac*	Ras-related C3 botulinum toxin substrate 1	2.37	2.52	1.97	1.84	2.51	2.04
*PI3K*	phosphoinositide-3-kinase				−4.06		
*AKT*	RAC serine/threonine-protein kinase				1.26		
*MAP2K1*	mitogen-activated protein kinase kinase 1	2.88	2.82	2.50	2.85	2.94	2.57
*MAP2K6*	mitogen-activated protein kinase kinase 6				−2.37		
*AP-1*	proto-oncogene protein c-fos		1.60		1.33		
*STAT1*	signal transducer and activator of transcription 1				2.03		
*IKBKE*	inhibitor of nuclear factor kappa-B kinase subunit epsilon				1.92		
RIG-I-like receptor (RLR) signaling pathway							
*RIG-I*	retinoic acid inducible gene I				2.32		
*LGP2*	laboratory of genetics and physiology 2				1.90		
*MDA5*	melanoma differentiation-associated gene 5				1.54		
*TRAF2*	TNF receptor-associated factor 2				2.19		
*MITA*	Mediator of IFN regulatory transcription factor 3 activation				1.29		
NOD-like receptor (NLR) signaling pathway							
*NLRP12*	NACHT, LRR and PYD domains-containing protein 12		−2.65		−2.05	−3.11	
*ASC*	apoptosis-associated speck-like protein containing a CARD				2.27		
* RIPK2 *	receptor-interacting serine/threonine-protein kinase 2				1.46		
* CARD8 *	caspase recruitment domain-containing protein 8				1.49		

**Table 5:** Fold changes of differentially expressed phagocytosis-related genes in the AS group and the IS group compared to the control.

Categories/ Gene Name	Description	Log_2_ (Fold Changes)					
		AS Group			IS Group		
		6 Hpi	24 Hpi	72 Hpi	6 Hpi	24 Hpi	72 Hpi
Internalization and formation of the phagosome							
*TLR4*	toll-like receptor 4				2.30	1.71	
*MR*	mannose receptor	−2.71	−1.43		−3.02		
*CR1*	complement receptor 1		1.69			2.15	
*αvβ5*	integrin αvβ5				−2.45		
*iC3b*	the fragment of complement component 3				−3.36		
*Collectin*	C-type lectin	−4.00	−1.66		−4.85		
*F-actin*	actin beta/gamma 1	3.60		−3.11	−3.66	−3.71	−2.46
Early phagosome							
*Rab5*	ras-related protein Rab-5B	2.47		1.84		1.82	
*vATPase*	V-type H^+^-transporting ATPase	2.21			−1.01	1.59	
*CALR*	calreticulin					2.10	
Mature phagosome							
*TUBA*	tubulin alpha				1.14	1.58	1.63
*TUBB*	tubulin beta	3.63	1.60		−1.21	2.28	2.34
*vATPase*	V-type H^+^-transporting ATPase	2.21			−1.01	1.59	
*Dynein*	dynein heavy chain 2				−1.82		
Phagolysosome							
*sec61*	protein transport protein SEC61 subunit beta				1.36		
*vATPase*	V-type H^+^-transporting ATPase	2.21			−1.01	1.59	
*NOS*	nitric-oxide synthase				−3.07		
*TAP*	ATP-binding cassette subfamily B member 3				2.17		
Activation of NADPH oxidase							
*gp91*	NADPH oxidase 1		1.62		1.57	1.40	
*p40phox*	neutrophil cytosolic factor 4				1.27		
*p47phox*	neutrophil cytosolic factor 1	2.75	2.32		2.53	2.04	1.52
*P67phox*	neutrophil cytosolic factor 2					1.40	
*Rac*	Ras-related C3 botulinum toxin substrate 1	2.37	2.52	1.97	1.84	2.51	2.04
Antigen presentation							
*MHC II*	MHC class II antigen	3.14	−1.87		−2.54		

**Table 6:** Fold changes of up-regulated apoptosis-related genes in the AS group and the IS group compared to the control.

Gene Name	Description	Log_2_ (Fold Changes)					
		AS Group			IS Group		
		6 Hpi	24 Hpi	72 Hpi	6 Hpi	24 Hpi	72 Hpi
*p53*	tumor protein p53				1.66		
*IP3R*	inositol 1,4,5-triphosphate receptor type 3				1.17		
*Perforin*	perforin 1	3.24			5.58		
*MERK2*	mitogen-activated protein kinase kinase 2	2.88	2.82	2.50	2.85	2.95	2.57
*Cathepsin*	cathepsin B		6.19	3.24	1.57	2.81	
*AP1*	proto-oncogene protein c-fos		1.60		1.33		
*GZMB*	granzyme B	1.22		1.57	5.59		5.34
*IL-3R*	cytokine receptor common subunit beta				2.56		
*TRAF12*	TNF receptor-associated factor 2	2.91			2.19		
*Fas*	tumor necrosis factor receptor superfamily member 6				2.34		
*TrkA*	neurotrophic tyrosine kinase receptor type 1				6.49		
*NIK*	mitogen-activated protein kinase kinase kinase 14				1.24		
*FLIP*	CASP8 and FADD-like apoptosis regulator				1.26		
*eiF2α*	translation initiation factor 2 subunit 1				1.25		
*Calpain*	calpain-1				1.49		
*ARTS*	septin 4				2.45		
*AIF*	apoptosis-inducing factor 1				1.40		
*Gadd45*	growth arrest and DNA-damage-inducible protein				2.32		
*ASK1*	mitogen-activated protein kinase kinase kinase 5					5.27	
*CytC*	cytochrome c					1.90	1.80

## Data Availability

The fastq files containing the raw reads obtained from each Illumina library in this study could be obtained from the Sequence Read Archive (SRA) under BioProject PRJNA781380. All the data are also available from the corresponding author upon reasonable request.

## Supplementary Materials

The following are available online at https://www.mdpi.com/article/10.3390/biology10111218/s1. Supplementary Table S1: Details of RNA-Seq data. L, liver; S, spleen; AL group, liver in active turtles; IL group, liver in inactive turtles; AS group, spleen in active turtles; IS group, spleen in inactive turtles. One library was constructed for liver and spleen tissue from the control group, while three libraries were constructed for liver and spleen tissue from AL, IL, AS, and IS groups at 6, 24, and 72 hpi (hours post-injection), except that only two libraries from IL group at 6 hpi because one library was failed in quality. In total, 37 libraries were constructed and sequenced. Supplementary Table S2: The list of up-regulated KEGG pathways in livers from active and inactive turtles compared to that in control group. Supplementary Table S3: The list of down-regulated KEGG pathways in livers from active and inactive turtles compared to that in control group. Supplementary Table S4: The list of up-regulated KEGG pathways in spleens from active and inactive turtles compared to that in control group. Supplementary Table S5: The list of down-regulated KEGG pathways in spleens from active and inactive turtles compared to that in control group. Supplementary Table S6: The sequence information of primers for qPCR in this study.

## Abbreviations

Akt, related to the A and C kinase; CCL, C-C motif chemokine; CFU, colony forming unit; CXCL, C-X-C motif chemokine; ECM, extracellular matrix; GAPDH, glyceraldehyde-3-phosphate dehydrogenase; IL, interleukin; KEGG, Kyoto Encyclopedia of Genes and Genomes; MHC, major histocompatibility complex; NADPH, nicotinamide adenine dinucleotide phosphate coenzyme II; NCBI, National Center for Biotechnology Information; NF-κB, nuclear factor kappa-B; NLR, nucleotide-binding domain-like receptor; NOD, nucleotide-binding domain; PCR, polymerase chain reaction; PI3K, phosphoinositide-3-kinase; PRR, pattern recognition receptor; RIG-I, retinoic acid-inducible gene I; RLR, retinoic acid-inducible gene I-like receptor; TLR, toll-like receptor; TNF, tumor necrosis factor; TNFSF, tumor necrosis factor ligand superfamily member. This list only addresses the abbreviations in contexts. The abbreviations in Table 1, Table 2, Table 3, Table 4, Table 5 and Table 6 have been explained separately.
