# Supplementary material for: Comparative Transcriptome Analysis Reveals the Molecular Immunopathogenesis of Chinese Soft-Shelled Turtle (Trionyx sinensis) Infected with Aeromonas hydrophila
Source: Biology (Basel). 2021 Nov 22;10(11):1218. doi: 10.3390/biology10111218 (PMC8615003; doi:10.3390/biology10111218)
Supplement: Supplementary file 1 [file biology-10-01218-s001.zip › Supplementary materials/Supplementary Table S1.pdf]

Supplementary Table S1. Details of RNA-Seq data.

Sample Name	Tissue	Hpi	Group	Clean reads	Clean bases	Mapping rate (%)	Q30 (%)
L0h	liver	0h	control	21,169,314	6,331,476,518	80.08	93.16
AL6h	liver	6h	active	25,543,078	7,636,810,006	82.51	93.98
AL24h	liver	24h	active	25,598,000	2,285,9624,798	83.53	93.76
AL72h	liver	72h	active	25,280,622	2,2681,617,450	82.52	93.50
IL6h	liver	6h	inactive	24,315,264	7,265,318,350	82.77	93.43
IL24h	liver	24h	inactive	27,987,993	8,363,911,033	81.17	93.27
IL72h	liver	72h	inactive	28,262,185	8,442,516,054	82.71	93.99
S0h	spleen	0h	control	23,303,214	6,960,655,810	81.67	93.23
AS6h	spleen	6h	active	24,511,961	7,330,036,160	80.14	93.72
AS24h	spleen	24h	active	21,683,974	6,456,128,289	82.50	93.63
AS72h	spleen	72h	active	26,401,102	7,560,539,150	82.32	93.62
IS6h	spleen	6h	inactive	29,571,593	8,841,001,329	80.59	93.68
IS24h	spleen	24h	inactive	29,501,061	8,810,760,554	79.65	93.27
IS72h	spleen	72h	inactive	29,617,004	8,822,691,248	82.59	93.92
