# Supplementary material for: Comparative Transcriptome Analysis Reveals the Molecular Immunopathogenesis of Chinese Soft-Shelled Turtle (Trionyx sinensis) Infected with Aeromonas hydrophila
Source: Biology (Basel). 2021 Nov 22;10(11):1218. doi: 10.3390/biology10111218 (PMC8615003; doi:10.3390/biology10111218)
Supplement: Supplementary file 1 [file biology-10-01218-s001.zip › Supplementary materials/Supplementary Table S6.pdf]

Supplementary Table S6. The sequence information of primers for qPCR.

Gene name	Forward primer（5'-3'）	Reverse primer（5'-3'）
IKBKE	CGATAGCCGCTTGTAACGTG	ACCTGGGCCAAATTCGGTTT
CXCL10	ACCAATCAGCGTCAAGGAGG	CTGCGAGAGGTTGAGGAGTG
IL1β	CTGGCTTGCAGAGACCGT	CAGCCTCACTTGGTGTTGGA
IL8	GCAAAGTCCTTGTTGCTGGG	TCTGTGTCAGCTTCACGTCC
IRF7	ATGGACTCTGGATGGAGGCT	AAGGAATCGGCTGTTCTGGG
MAP2K6	TTCCGGCAGACAAGTTCTCC	GCCACGTCTGTCTCTTTGGA
CXCL11	CCCTCTGAACGATACACTCCA	TGCCATTCCTTGCAGCAGAT
MyD88	TGTGTCTTGCAGAAGAGGAC	CATCAAACAGCTCTGGCGTG
TLR8	CCCCTACCCATGTGAACCTT	GAGAGGTGCTGGAGAGGTTT
